# Threat, hostility and violence in childhood and later psychotic disorder: population-based case–control study

**DOI:** 10.1192/bjp.2020.133

**Published:** 2020-10

**Authors:** Craig Morgan, Charlotte Gayer-Anderson, Stephanie Beards, Kathryn Hubbard, Valeria Mondelli, Marta Di Forti, Robin M. Murray, Carmine Pariante, Paola Dazzan, Thomas J. Craig, Ulrich Reininghaus, Helen L. Fisher

**Affiliations:** 1 Institute of Psychiatry, Psychology & Neuroscience, King's College London; and National Institute for Health Research (NIHR) Mental Health Biomedical Research Centre, South London and Maudsley NHS Foundation Trust and King's College London; and Economic and Social Research Council (ESRC) Centre for Society and Mental Health, King's College London, UK; 2 Institute of Psychiatry, Psychology & Neuroscience, King's College London; and Economic and Social Research Council (ESRC) Centre for Society and Mental Health, King's College London, UK; 3 Institute of Psychiatry, Psychology & Neuroscience, King's College London, UK; 4 Institute of Psychiatry, Psychology & Neuroscience, King's College London; and National Institute for Health Research (NIHR) Mental Health Biomedical Research Centre, South London and Maudsley NHS Foundation Trust and King's College London, UK; 5 FRS, Institute of Psychiatry, Psychology & Neuroscience, King's College London; and National Institute for Health Research (NIHR) Mental Health Biomedical Research Centre, South London and Maudsley NHS Foundation Trust and King's College London, UK

**Keywords:** Childhood experience, psychotic disorders, trauma, schizophrenia, aetiology

## Abstract

**Background:**

A growing body of research suggests that childhood adversities are associated with later psychosis, broadly defined. However, there remain several gaps and unanswered questions. Most studies are of low-level psychotic experiences and findings cannot necessarily be extrapolated to psychotic disorders. Further, few studies have examined the effects of more fine-grained dimensions of adversity such as type, timing and severity.

**Aims:**

Using detailed data from the Childhood Adversity and Psychosis (CAPsy) study, we sought to address these gaps and examine in detail associations between a range of childhood adversities and psychotic disorder.

**Method:**

CAPsy is population-based first-episode psychosis case–control study in the UK. In a sample of 374 cases and 301 controls, we collected extensive data on childhood adversities, in particular household discord, various forms of abuse and bullying, and putative confounders, including family history of psychotic disorder, using validated, semi-structured instruments.

**Results:**

We found strong evidence that all forms of childhood adversity were associated with around a two- to fourfold increased odds of psychotic disorder and that exposure to multiple adversities was associated with a linear increase in odds. We further found that severe forms of adversity, i.e. involving threat, hostility and violence, were most strongly associated with increased odds of disorder. More tentatively, we found that some adversities (e.g. bullying, sexual abuse) were more strongly associated with psychotic disorder if first occurrence was in adolescence.

**Conclusions:**

Our findings extend previous research on childhood adversity and suggest a degree of specificity for severe adversities involving threat, hostility and violence.

Research into the relationship between childhood adversities and later psychosis, broadly defined, has grown in recent years.^1^ The findings are consistent: most forms of adversity, including bullying, family breakdown, neglect and abuse, are associated with a two- to fourfold increased likelihood of psychosis.^2^ However, there remain several gaps and unanswered questions.

The most methodologically robust studies (i.e. prospective designs, large samples, etc.) have been of low-level psychotic experiences in general population samples.^2,3^ These experiences frequently co-occur with symptoms of depression and anxiety and with suicidality, and have an indeterminate association with subsequent psychotic disorder.^4^ Certainly, the majority of people who report low-level psychotic experiences do not develop a psychotic disorder. Consequently, the extent to which associations reported in these studies can be extrapolated to psychotic disorders is uncertain. There are fewer studies specifically on childhood adversity and psychotic disorder and, with some exceptions (e.g. ref. ^5^), these have tended to be on smaller samples, either have no or a highly select control group and have rarely adjusted for potential confounders, such as family history of psychosis (proxy genetic risk) and parental social class.^1^ There is, then, a need for more methodologically robust studies to further examine the nature and strength of associations between childhood adversities and psychotic disorder.

Further, studies have so far tended to focus on the effect of individual adversities, with exposure categorised simply into present at any point during childhood or not, with relatively low thresholds for ratings of present.^1^ However, individuals are often exposed to multiple interrelated adversities, and the timing, severity and type of exposure are important in relation to other mental disorders. There is some evidence, for example, that events involving loss, humiliation and entrapment are particularly important in the onset of depression^6^ and it has been hypothesised that events involving severe interpersonal threat and hostility may have specific effects on risk of psychosis,^7^ possibly via cognitive and affective pathways.^1^ However, with some exceptions, these dimensions have not been examined in relation to psychoses.

We established the Childhood Adversity and Psychosis (CAPsy) study to address these gaps and examine in detail associations between a range of childhood adversities and psychotic disorder, focusing on type (i.e. household discord, psychological abuse, physical abuse, sexual abuse, bullying), age, frequency and severity of exposure, on interactions with other risk factors (e.g. adult adversities, substance use) and with putative psychological and biological mechanisms. In this paper, we report findings from our primary analyses, in which we sought to test the hypotheses that:

(a) each adversity (i.e. household discord, psychological abuse, physical abuse, sexual abuse, bullying) is associated with increased odds of psychotic disorder
(b) there is a linear association between number of types of adversity experienced and odds of psychotic disorder
(c) odds of psychotic disorder are greatest for those who report (i) early (i.e. age under 11 years), (ii) frequent (i.e. at least weekly) and (iii) severe (i.e. involving extreme threat, hostility, violence) exposure.

## Method

### Design

The Childhood Adversity and Psychosis (CAPsy) study is a population-based case–control study of first-episode psychosis, conducted over a four-year period (2010–2014).

### Sample 1: cases

Inclusion criteria for cases: were age 18–64 years; resident within defined catchment areas in south-east London, UK; presence of a first-episode psychotic disorder (i.e. ICD-10 diagnoses F20–29 and F30–33 (with psychotic symptoms, i.e. affective psychoses)) within the time frame of the study; and no previous contact with mental health services for psychosis. Exclusion criteria were: evidence that psychotic symptoms were precipitated by an organic cause; transient psychotic symptoms resulting from acute intoxication as defined by ICD-10; severe intellectual disabilities; and insufficient understanding of English to complete assessments.

To identify potential cases, a team of researchers screened, at least weekly, general and specialist in-patient, out-patient and community services in the catchment areas. That is, researchers liaised with designated clinical staff within each service to review new referrals and admissions to identify potentially eligible individuals. All potential cases were screened for inclusion using the Screening Schedule for Psychosis.^8^ All who met the inclusion criteria were approached and informed consent was sought. We were not able to collect any information on those who could not be contacted or who refused. However, we were able to compare the basic characteristics of consenting cases with those from a concurrent case-register-based incidence study of all individuals with a first-episode psychosis in our catchment areas and a previous incidence study in these areas.

### Sample 2: controls

A population-based and demographically representative sample of controls resident in our catchment areas, aged 18–64 years and without a current or past history of psychotic disorder, was recruited using a mixture of quota and random sampling. First, quotas were set for gender, age group and ethnic group. The quotas for each group were set to ensure recruitment of a sample that reflected the demographic profile, based on the 2011 UK census, of the local population and that included a sufficient number of controls from Black Caribbean and Black African groups for analyses by ethnic group. Second, two sampling frames were used to identify and recruit controls to fill these quotas: (a) the UK postal address file and (b) general practitioner (GP) lists (see supplementary Appendix, available at https://doi.org/10.1192/bjp.2020.133, for more detail on control recruitment).

### Data collection

All cases and controls completed a series of interviews and assessments that elicited information on a wide range of clinical, social, neurocognition, social cognition and biological variables.

### Childhood adversity

Data on childhood adversities before age 17 years were collected using sections of the Childhood Experience of Care and Abuse (CECA) schedule,^9^ an in-depth face-to-face semi-structured interview, and an adapted version of the Bullying Questionnaire.^10,11^ In this paper, we focus on household discord, psychological abuse, physical abuse, sexual abuse and bullying. The CECA has a high degree of interrater reliability^9^ and reasonable levels of validity^12^ and the Bullying Questionnaire has high levels of test–retest reliability.^10^ We used life-course interview techniques, including anchoring by key dates, to aid recall. The same rating scales were used in the CECA and Bullying Questionnaire to capture severity and frequency of experiences, and age at first exposure. All ratings were made by consensus, based on reports elicited in interviews. Severity was rated on a four-point scale: none, some, moderate and marked, with the exception of household discord, which included an additional point to capture domestic violence. Ratings of ‘none’ and ‘some’ were combined into a reference category of ‘absent’, in line with the CECA manual. Frequency was rated as never, rare (once or twice), occasional (more than twice, less than monthly), frequent (monthly) or very frequent (weekly), and dichotomised for analyses into frequent (monthly or more often) versus other (less than monthly). Age at exposure was defined as age at first occurrence of adversity and dichotomised for analyses into 0–11 years old (childhood) and 12–16 years old (adolescence). In addition, a rating of doubt (i.e. none versus some) was made for each interview to capture any uncertainty about the veracity of responses.

### Demographic, clinical and other data

An extended Medical Research Council Sociodemographic Schedule was used to collect data on demographic characteristics, social circumstances and relationships, and social class of parents during childhood according to the European Socio-Economic Classification system (https://www.iser.essex.ac.uk/archives/esec). For analyses, main parent's (i.e. head of household's) social class during childhood was grouped into three classes: salariat, intermediate, working class. In this system, students and long-term unemployed are considered non-classifiable.

Symptoms were assessed using the Schedules for Clinical Assessment in Neuropsychiatry (SCAN).^13^ Information from the SCAN and clinical records was used to complete the Operational Criteria Checklist for Psychotic and Affective Disorders (OPCRIT),^14^ from which we derived DSM-IV and ICD-10 diagnoses for cases. Diagnoses were dichotomised into non-affective and affective psychoses. The Nottingham Onset Schedule^15^ was used to estimate date of onset of psychosis, defined as the first point when there was clear evidence of clinically meaningful psychotic symptoms, operationalised as a score of at least two for a psychosis item on Rating Scale 2 of the SCAN.^13^

The Family Interview for Genetic Studies (FIGS)^16^ was used to collect information on participants’ family history of mental illness. For analyses, parental history of psychosis was used as proxy for genetic risk.

### Ethics

All procedures contributing to this work comply with the ethical standards of the relevant national and institutional committees on human experimentation and with the Helsinki Declaration of 1975, as revised in 2008. All procedures involving human participants were approved by the South London and Maudsley NHS Foundation Trust and the Institute of Psychiatry Research Ethics Committee (ref: 321/05, including amendments 1 to 9). All participants provided written informed consent.

### Analysis

We used logistic regression to estimate odds ratios with 95% confidence intervals. We began by estimating main effects (odds ratios) for each form of adversity, dichotomised into absent (none, some) and present (moderate, marked). We then extended these analyses in two ways. First, we created a simple index of childhood adversity, counting the number of different types of exposure that participants reported, to examine whether there was evidence of a linear relationship with psychosis. Second, we interrogated each exposure in more detail, examining variations in effect by type, age at first experience, frequency and severity. All analyses were adjusted for putative confounders and weighted to take account of the oversampling of Black Caribbean and Black African controls. Finally, we repeated all analyses including only those cases and controls for whom there was no rating of doubt for CECA interviews. All analyses were conducted in Stata version 15 for Windows.

## Results

### Sample

During the study period, we identified, consented and assessed 374 individuals with a first-episode psychosis (62.4% of 599 potential cases identified) and 301 population-based controls (133 via the postal address file; 168 via GP lists). The demographic characteristics of controls in our sample (after weighting) were similar to those of the local population (supplementary Table 1). The demographic and clinical characteristics of cases in our sample were similar to those for other previous and concurrent incidence studies (supplementary Table 2). Compared with controls, cases were younger, more often men and more often of Black Caribbean and Black African ethnicity, reflecting what we know about the demographic characteristics of first-episode psychosis in south London (Table 1). In total, 342 cases (91%) and 297 controls (99%) completed at least part of the CECA interview. There were no clear differences by age, gender or ethnic group between cases who did and did not complete a CECA interview (supplementary Table 3). Of the 342 cases, 17 had developed psychosis during childhood (i.e. before age 17 years) and were excluded from analyses.

**Table 1:** Sociodemographic and clinical characteristics by case-control status

	Controls, *n* = 301		Cases, *n* = 374				
	Mean (s.d.)	Mean (s.d.)	*t*	d.f.	*P*
Age, years	35.3 (12.3)		28.9 (8.9)		7.84	673	<0.001
	*n* (weighted %)		*n* (weighted %)		χ²	d.f.	*P*
Gender							
Men	153	(50.1)	229	(61.2)	7.34	1	0.007
Women	148	(49.9)	145	(38.8)			
Ethnicity							
White British	131	(42.6)	92	(24.9)	39.85	5	<0.001
White non-British	44	(21.8)	46	(12.4)			
Black Caribbean	44	(11.2)	60	(16.2)			
Black African	50	(13.0)	94	(25.4)			
Asian (all)	17	(6.8)	23	(6.2)			
Other	15	(4.7)	55	(14.9)			
Economic status, at interview^a^							
Employed	194	(68.0)	76	(22.1)	147.02	2	<0.001
Unemployed	43	(13.0)	195	(56.7)			
Economically inactive (including students)	64	(19.0)	73	(21.2)			
Parental social class^b^							
Salariat	157	(52.8)	95	(33.2)	31.88	2	<0.001
Intermediate	87	(29.2)	88	(30.8)			
Working class	52	(18.0)	103	(36.0)			
Parental psychosis^c^							
No	262	(95.9)	265	(88.6)	17.98	1	<0.001
Yes	6	(4.1)	34	(11.4)			
Diagnosis							
Schizophrenia	–	–	180	(49.1)	–	–	–
Other non-affective psychosis^d^	–	–	112	(29.9)	–	–	–
Affective psychosis	–	–	82	(21.9)	–	–	–

a. Missing: 30 (7%).

b. Unclassified (i.e. long-term unemployed; student): 4; missing: 89 (13%).

c. Missing: 108 (16%).

d. Includes schizoaffective disorder, delusional disorder and psychosis not otherwise specified (which includes 23 with insufficient information to derive an Operational Criteria Checklist for Psychotic and Affective Disorders diagnosis).

### Main effects: overall

Reports of moderate and marked adversity were high, especially among cases, and each form of childhood adversity was associated with increased odds of psychosis, independent of age, gender and ethnicity (Table 2). The magnitude of adjusted odds ratios varied, ranging from 1.43 for bullying to 3.95 for psychological abuse. These effects were broadly similar, albeit with some variation, for non-affective and affective psychoses (supplementary Table 4), for men and women (supplementary Table 5) and for younger (under age 30) and older (age 30 and over) participants (supplementary Table 6).

**Table 2:** Main effects for each type of childhood adversity^a^

	Controls (*n* = 297), *n* (%)^c^	Cases (*n* = 325), *n* (%)^c^	Unadjusted OR	95% CI	*P*	Adjusted OR^b^	95% CI	*P*
Household discord^d^										
No	184	(62.2)	137	(51.9)	1.00	–	–	1.00	–	–
Yes	112	(37.8)	127	(48.1)	1.58	1.10–2.26	0.013	1.68	1.14–2.48	0.009
Psychological abuse^e^										
No	283	(95.9)	226	(85.6)	1.00	–	–	1.00	–	–
Yes	12	(4.1)	38	(14.4)	4.28	2.08–8.87	<0.001	3.95	1.80–8.66	0.001
Physical abuse^f^										
No	234	(79.1)	196	(64.5)	1.00	–	–	1.00	–	–
Yes	62	(20.9)	108	(35.5)	2.36	1.59–3.51	<0.001	2.28	1.44–3.62	<0.001
Sexual abuse^g^										
No	273	(93.5)	245	(84.8)	1.00	–	–	1.00	–	–
Yes	19	(6.5)	45	(15.2)	2.59	1.39–4.82	0.003	2.58	1.40–4.76	0.002
Bullying^h^										
No	207	(70.4)	176	(62.2)	1.00	–	–	1.00	–	–
Yes	87	(29.6)	107	(37.8)	1.63	1.12–2.35	0.010	1.43	0.97–2.10	0.070

a. All analyses are weighted to account for oversampling of Black Caribbean and Black African controls.

b. Adjusted for age, gender and ethnicity.

c. Percentages are for cases and controls with complete data; cases with childhood onset are excluded.

d. 62 missing (1 control, 61 cases).

e. 63 missing (2 controls, 61 cases).

f. 22 missing (1 control, 21 cases).

g. 40 missing (5 controls, 35 cases).

h. 45 missing (3 controls, 42 cases).

When we repeated analyses on those with complete data on additional putative confounders (240 cases; 264 controls), we found that the effects were a bit lower than what we observed in the full sample, with some attenuation (most notably for physical abuse) when adjusted for parent history of psychosis and parent social class (supplementary Table 7). Further, when we restricted analyses to those for whom there were no doubts about the possible validity of responses to the CECA questions, the adjusted odds ratios were similar to those observed in the full sample (supplementary Table 8).

### Age and frequency

There was some tentative evidence of variations in effects by age at first reported exposure to adversity. For example, odds ratios were higher when first occurrence was in adolescence (versus childhood) for sexual abuse (adjusted OR = 6.4 *v*. 2.4), bullying (adj. OR = 1.8 *v*. 1.2) and physical abuse (adj. OR = 3.6 *v*. 2.1) (Table 3; supplementary Table 9), albeit we cannot exclude the possibility that these patterns reflect sampling variation. Effects for household discord and psychological abuse were similar for both developmental periods. We found no strong evidence that effects varied by frequency (supplementary Table 10).

**Table 3:** Childhood adversities and psychotic disorder, overall and by age: summary^a^,^b^

	Main effect		By age at first occurrence				By severity					
	Overall, adjusted OR^c^ (95% CI)	0–11 years, adjusted OR^c^ (95% CI)	12–16 years, adjusted OR^c^ (95% CI)	Moderate, adjusted OR^c^ (95% CI)	Marked, adjusted OR^c^ (95% CI)	Domestic violence, adjusted OR^c^ (95% CI)
Household discord	1.68	(1.14–2.48)	1.68	(1.10–2.55)	1.70	(0.83–3.48)	0.87	(0.50–1.52)	1.22	(0.64–2.31)	4.40	(2.37–8.16)
Psychological abuse	3.95	(1.80–8.66)	4.15	(1.65–10.42)	3.15	(0.80–12.36)	3.45	(1.35–8.82)	5.60	(1.47–21.36)	–	–
Physical abuse	2.28	(1.44–3.62)	2.07	(1.24–3.44)	3.56	(1.42–8.93)	2.07	(1.27–3.39)	3.97	(1.59–9.89)	–	–
Sexual abuse	2.58	(1.40–4.76)	2.42	(1.21–4.82)	6.39	(1.68–24.29)	1.58	(0.65–3.88)	3.73	(1.63–8.54)	–	–
Bullying	1.43	(0.97–2.10)	1.19	(0.75–1.90)	1.75	(1.05–2.96)	1.27	(0.82–1.95)	1.91	(1.00–3.64)	–	–
Any adversity	2.01	(1.31–3.08)	1.95	(1.25–3.05)	2.26	(1.24–4.12)	1.28	(0.80–2.06)	3.85	(2.27–6.54)	–	–

a. Note: All analyses are weighted to account for oversampling of Black Caribbean and Black African controls.

b. See supplementary Tables 9 and 13 for full data.

c. Adjusted for age, gender, and ethnicity.

### Cumulative effects

**Fig. 1:**
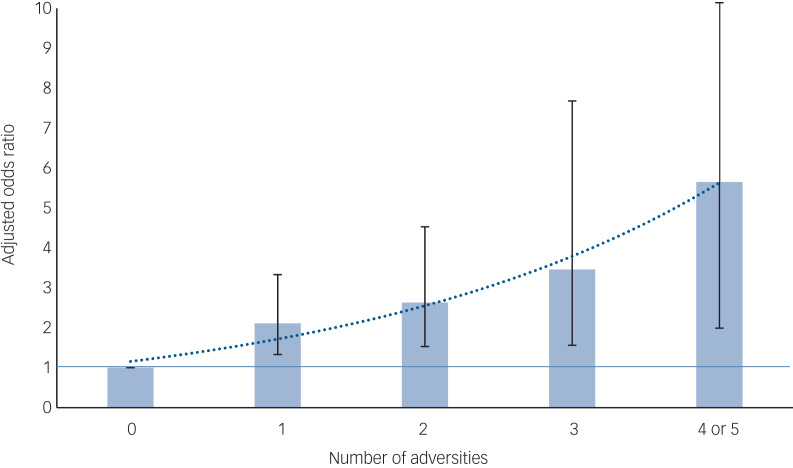
Association between number of adversities and psychotic disorder: adjusted odds ratios with 95% confidence intervals (see supplementary Table 1 for full data).

Most forms of childhood adversity were, to varying degrees, associated with each other among both cases and controls (supplementary Table 1^1^). However, among controls associations were, overall, weaker and sexual abuse was associated only with physical abuse. Compared with controls, cases were progressively more likely to report exposure to a greater number of adversities (Fig. 1; supplementary Table 1^2^). For every additional adversity, the odds of psychosis increased by, on average, around 50%.

The 95% CI line for four or five adversities is truncated, with upper limit: 16.08. Number of adversities entered as a continuous variable: adjusted OR = 1.53 (95% CI 1.27–1.84); for each additional adversity, odds of psychotic disorder increase by, on average, around 50%. Linear versus quadratic term likelihood ratio test: χ^2^ = 1.11, P = 0.29. All analyses are weighted to account for oversampling of Black Caribbean and Black African controls, and adjusted for age, gender and ethnicity.

### Severity

When we further examined effects by severity, we found strong evidence that the effects were greatest for the most severe forms of adversity and abuse (Table 3; supplementary Table 13). For household discord and bullying, only the most severe level of exposure was associated with increased odds of psychosis. For example, there was no evidence of an association for household discord, however severe, that did not include domestic violence. There was, however, a strong and large association for reports of domestic violence in the household during childhood and adolescence (adj. OR = 4.4; 95% CI 2.4–8.2). Similarly, for bullying, an effect was evident only for the most severe form of bullying, which invariably involved physical assault (adj. OR = 1.9; 95% CI 1.0–3.6). For the most severe forms of psychological, physical and sexual abuse, the odds ratios ranged from 3.7 to 5.6. To further illustrate the differences, around 42% (*n* = 99) of cases reported exposure to at least one form of severe adversity compared with around 16% (*n* = 46) of controls (adj. OR = 3.80; 95% CI 2.23–6.48) (Table 3). For any non-marked adversity (in the absence of a marked adversity), there was no strong evidence of a difference between cases and controls (adj. OR = 1.30, 95% CI 0.81–2.10).

## Discussion

We found strong evidence that several forms of childhood adversity were associated, to varying degrees, with increased odds of psychotic disorder in adulthood and that exposure to multiple adversities was associated with a linear increase in odds of psychosis. These associations remained robust when adjusted for putative confounders and when we sought to account for any doubt in reports of adversities. This provides perhaps the strongest evidence to date that associations between childhood adversities and broad psychosis phenotypes do extend to psychotic disorders. Our findings go further and, in considering more fine-grained aspects of adversity, suggest potential refinements to our understanding of the relationships between early adversity and psychosis, notably: severe forms of adversity, involving threat, hostility and violence, may specifically increase risk, and – more tentatively – for some experiences (e.g. bullying, sexual abuse), occurrence in adolescence may be especially important.

### Methodological issues

Our findings need to be considered in light of several potential limitations. First, differential recall of exposure to early adversity between cases and controls may create spurious associations, a problem that has been well rehearsed in relation to studies of abuse and psychosis.^17^ In our study, we sought to minimise this, first, by using a validated semi-structured interview, with life-course techniques to anchor memories, that elicited concrete and detailed descriptions of past experiences and, second, by making all ratings by consensus based on accounts elicited in interview. Positive ratings required clear descriptions of experiences. We further sought to assess the possible influence of recall bias by conducting a sensitivity analysis excluding all for whom there was any doubt, for whatever reason (e.g. current mental state), about the veracity of responses. Our findings remained robust. Further, in separate analyses comparing CECA data with self-report questionnaire data, we found no evidence that the validity of retrospective reports of abuse differed between cases and controls,^18^ and we have previously shown that reports of adversity among samples with a psychotic disorder remain stable over time.^19^ It is notable, moreover, that associations were strongest for the most severe forms of adversity, i.e. those that are, sadly, unlikely to be forgotten. This said, in some instances, experiences of adversity may still not have been captured in retrospective reports^20^ and this urges further caution in interpreting our findings.

Second, it is possible that potential controls who experienced early adversity were particularly reluctant to participate, leading to an underestimation of the prevalence of adversities in the population at risk. By using a mixture of quota and random sampling, we were, to a degree, able to maximise the representativeness of the control sample, which is an advance on previous studies. The addition of quotas ensures that, demographically at least, participants resemble the population at risk. However, potential participants were, of course, informed that the study was about childhood adversity and we consequently cannot rule out bias that may have arisen because of this.

Third, although our sample – given the depth of data – is larger than in previous studies, it is still relatively small and does not allow us to fully interrogate the interrelationships between the dimensions of type, age at first exposure, frequency and severity. When stratified by two or more of these dimensions, the number of participants in relevant cells was small. This points to a general problem: the challenge of balancing depth and breadth in studies of social contexts, experience and psychosis.

Finally, we were able to adjust analyses for important potential confounders, including proxy genetic risk. However, our measures of these were crude and, again, relied on participant self-report. Some residual confounding is consequently inevitable.

### Main effects

These limitations noted, our study provides strong evidence that associations between various forms of childhood adversity and broad psychosis phenotypes extend to psychotic disorder. Some of the methodologically stronger earlier studies of psychotic disorder were equivocal and findings mixed. For example, in our analyses of data from the ÆSOP study, we found modest associations, among women only, between sexual and physical abuse and psychotic disorder (i.e. a roughly twofold increased odds).^21^ In a subsequent study, we again found, at most, weak evidence of an association between physical (OR = 1.5) and sexual (OR = 1.8) abuse and psychotic disorder.^22^ We did, however, find evidence of a strong association with bullying (OR = 3.4).^23^ These inconsistencies may reflect methodological differences in measures used (including how presence and absence are defined), adversities considered and sample sizes.

### Clustering of adversities: cumulative effects

Specific forms of adversity rarely occur in isolation and several previous studies have examined linear associations between the number of adversities experienced and odds of psychosis.^1,2^ The evidence for cumulative effects of multiple adversities on odds of psychotic experiences is largely consistent.^2^ However, the evidence for psychotic disorder is less consistent. Our findings are clear and do suggest cumulative effects for those exposed to multiple adversities, a conclusion underscored by the observation that associations between the various adversities were stronger among cases than controls. This was particularly notable for sexual abuse. Put crudely, among cases, but not controls, sexual abuse frequently occurred against a background of household discord, other abuses and peer bullying.

### Specificity: type, age, severity

All forms of childhood adversity are associated with a wide range of subsequent adverse outcomes, including mental health problems broadly,^24^ physical health problems^25^ and problematic behaviours (e.g. substance use, conduct problems).^26^ It is possible that adversity has non-specific effects, increasing the risk of a range of disorders and adverse outcomes through general effects on stress response and other biological systems, with other factors influencing the form that problems take. At the same time, it is possible that there is some degree of specificity related to type, age at first exposure and severity.

Our findings, for example, tentatively suggest that age at first exposure may be important. We initially hypothesised that earlier exposure would have the strongest effects. However, in so far as there were any differences by age at first exposure, our findings point to adolescence as being especially important. Similarly, Cutajar et al^27^ found that sexual abuse after age 12 was associated with the greatest odds of psychotic disorder. This is plausible. Adversities that have a direct impact on the developing identity and sense of self (such as bullying or sexual abuse) during a period of considerable brain plasticity and when young people are acutely sensitive to peer influence and comparison may have especially strong effects. Indeed, this makes more sense still, if – as some suggest – psychoses are disorders of the self.^28^

Our findings more strongly suggest that severity of exposure is important. The cumulative effects noted above may, in part, capture overall severity, indexing exposure to linked adversities that together constitute a traumatic and chaotic childhood. Further, the defining features of severe adversities, as measured in our study, are severe threat, hostility and violence. Findings from several other studies fit with this. For example, a study by Arseneault et al^29^ found that bullying and maltreatment, but not accidents, between ages 5 and 12 years, were associated with increased odds of psychotic experiences at age 12. This further ties in with the findings already noted in the study by Cutajar et al, that the strongest effects were for the most severe forms of adversity and abuse (e.g. penetrative sexual abuse).^27^ In parallel work, analyses of data on adult life events from the CAPsy study suggest that intrusive events (i.e. events, such as physical assault, that involve unwanted intrusion into personal space) have the strongest effect on odds of psychotic disorder.^30^ Taken together, these findings suggest that it is the more severe forms of adversity that involve direct threat, hostility and violence, particularly during key developmental stages, that are important in the emergence of psychotic disorders.

### Mechanisms

Moreover, this interpretation fits with prominent theories and developing evidence on interrelated cognitive, affective and biological mechanisms that may link adversity and the development of psychotic disorder. For example, it is possible that experiences of threat, hostility and violence, particularly in adolescence, when beliefs about the self and the world crystallise, contribute to the development of cognitive biases and affective processes specifically linked to psychotic experiences, particularly paranoid delusions.^31^ It is further possible – though speculative – that emerging paranoia and other symptoms may lead to isolation and increase the risk of exposure to additional threats and adversities, constituting a vicious spiral that leads to disorder. Further, trauma is associated with an increased likelihood of dissociation, which has been implicated in the development of hallucinations.^32^ It further follows that, if there is some specificity between particular adverse experiences and individual psychotic experiences, we would expect those who endure multiple severe and co-occurring adversities to present with multiple distressing psychotic experiences in several domains (i.e. hallucinations, delusions, etc.); that is, with what we currently classify as psychotic disorder.

In addition, our findings fit with evidence that various forms of adversity have an impact on biological systems implicated in the underlying pathophysiology of psychoses, including increased amygdala volume^33^ and dysregulation of the hypothalamic–pituitary–adrenal (HPA) axis^34^ and dopamine system.^35^ More specifically, it is notable that both the amygdala and dopamine system are involved in the regulation of emotion responses, including fear, and the attachment of salience to external stimuli. It is possible, then, that prolonged exposure to severe threat, hostility and violence contribute to long-term changes in biological systems that underpin the cognitive and affective processes noted above: i.e. fear in response to, and attachment of, aberrant levels of salience to relatively neutral contexts and experiences, leading to the development of excessive suspiciousness and, ultimately, delusions of paranoia and reference and other psychotic symptoms.

### Implications

Our findings extend previous research on childhood adversity and suggest a degree of specificity for severe adversities involving threat, hostility and violence. There is a certain face validity to this. In so far as experiences across the life course directly increase risk of psychotic disorders – which are relatively rare, serious and often long-lasting – it seems plausible that it will be extreme and severe experiences that do so. This conclusion has important implications for mental health services. It suggests that packages of care should be tailored to specific sociodevelopmental trajectories, taking into account the life histories and social circumstances of patients and drawing from an eclectic mix of interventions, from the social to the pharmacological.

## Data Availability

The data that support the findings of this study are available from the corresponding author, C.M., on reasonable request.

## Supplementary material

For supplementary material accompanying this paper visit https://doi.org/10.1192/bjp.2020.133. click here to view supplementary material
