# Supplementary material for: Threat, hostility and violence in childhood and later psychotic disorder: population-based case–control study
Source: Br J Psychiatry. 2020 Oct;217(4):575–82. doi: 10.1192/bjp.2020.133 (PMC7525109; doi:10.1192/bjp.2020.133)
Supplement: Supplementary file 1 [file S0007125020001336sup001.zip › S0007125020001336sup001.docx]

**Supplementary Materials (1) Tables and Figures**

**Supplementary Table 1.** Comparisons of CAPsy control sample with population at risk in the catchment area, using 2011 UK census information.

|  | 2011 UK Census for study catchment area^a^  n (%) | CAPsy control sample  n (weighted %) |
| --- | --- | --- |
| **Total samples** ^b^ | 426,460 | 301 |
| **Sex**  Men | 212,984 (49.9) | 153 (50.1) |
| Women | 213,476 (50.1) | 148 (49.9) |
| **Ethnic group**  White British | 175,706 (41.2) | 131 (42.6) |
| White non-British | 80,459 (18.9) | 44 (21.8) |
| Black-Caribbean | 43,894 (10.3) | 44 (11.2) |
| Black-African | 53,835 (12.6) | 50 (13.0) |
| Asian or Asian British | 37,216 (8.7) | 17 (6.8) |
| Mixed ^c^ | 22,677 (5.3) | - |
| Other | 12,673 (2.9) | 15 (4.7) |
| **Age groups**  18-29 | 145,414 (34.1) | 112 (31.1) |
| 30-49 | 208,834 (49.0) | 144 (52.8) |
| 50-64 | 72,227 (16.9) | 45 (16.1) |
|  |  |  |
| **In employment** | 314,656 (73.8) | 194 (68.0) |

^a^ South-east London Boroughs of Lambeth and Southwark; data are provided by the UK Office for National Statistics

^b^ Frequencies may not add up to 100% due to missing values; percentages are weighted

^c^ Mixed ethnicity not specified as a category in the CAPsy study; mixed ethnicity is included in the Other ethnic category.

**Supplementary Table 2.** Comparison of sociodemographic and clinical characteristics of CAPsy cases and concurrent and previous incidence studies.

|  | | CAPsy  n, 374 | | Oduloa et al 2019**  n, 558 | | AESOP, London  n, 308* | |
| --- | --- | --- | --- | --- | --- | --- | --- |
| Age | |  |  |  |  |  |  |
|  | Mean | 29.2 | | 33.3 | | 30.4 | |
|  | sd | 8.9 | | 10.7 | | 11.2 | |
|  |  | n (%) | | n (%) | | n (%) | |
| Sex | |  |  |  |  |  |  |
|  | Men | 205 | (61.7) | 292 | (52.3) | 175 | (56.8) |
|  | Women | 127 | (38.3) | 266 | (47.7) | 133 | (43.2) |
| Ethnicity | |  |  |  |  |  |  |
|  | White British | 88 | (26.5) | 133 | (23.8) | 81 | (26.3) |
|  | White non-British | 45 | (13.6) | 75 | (13.4) | 30 | (9.7) |
|  | Black Caribbean | 55 | (16.6) | 91 | (16.3) | 104 | (33.8) |
|  | Black African | 83 | (25.0) | 147 | (26.3) | 63 | (20.5) |
|  | Asian (all) | 15 | (4.5) | 44 | (7.9) | 10 | (3.3) |
|  | Other | 46 | (13.9) | 68 | (13.1) | 20 | (6.5) |
| Economic status | |  |  |  |  |  |  |
|  | Employed | 64 | (21.1) | 99 | (19.6) | 75 | (24.8) |
|  | Unemployed | 173 | (56.9) | 346 | (68.5) | 177 | (58.6) |
|  | Economically inactive | 67 | (22.0) | 60 | (11.9) | 50 | (16.6) |
| Parental social class | |  |  |  |  |  |  |
|  | Salariat | 95 | (24.4) | - | - | - | - |
|  | Intermediate | 88 | (23.5) | - | - | - | - |
|  | Woking class | 103 | (27.5) | - | - | - | - |
|  | Non-classifiable, Missing | 88 | (23.5) | - | - | - | - |
| Parental psychosis | |  |  |  |  |  |  |
|  | No | 237 | (90.1) | - | - | 156 | (84.8) |
|  | Yes | 26 | (9.9) | - | - | 28 | (15.2) |
| Diagnosis | |  |  |  |  |  |  |
|  | Schizophrenia | 135 | (40.7) | - | - | 154 | (50.0) |
|  | Other non-affective psychosis† | 132 | (39.8) | - | - | 75 | (24.3) |
|  | Affective psychosis | 65 | (19.5) | - | - | 79 | (25.7) |

* Total baseline AESOP incidence sample for London only

** Oduola, S., Das-Munshi, J., Bourque, F., Gayer-Anderson, C., Tsang, J., Murray, R., Craig, T., & Morgan, C. (submitted) Incidence of first episode psychosis among minority ethnic groups: Findings from the Clinical Record Interactive Search – First-Episode Psychosis Study

**Supplementary Table 3.** Comparison of cases with and without complete CECA.

|  | | Cases with CECA  n = 342 | | Cases without CECA  n = 32 | | t | df | p |
| --- | --- | --- | --- | --- | --- | --- | --- | --- |
| Age | |  |  |  |  |  |  |  |
|  | Mean | 31.7 | | 32.4 | | -0.38 | 673 | 0.706 |
|  | sd | 11.1 | | 11.0 | |  |  |  |
|  |  | n (%) | | n (%) | | χ² | df | p |
| Sex | |  |  |  |  |  |  |  |
|  | Men | 213 | (62.3) | 16 | (50.0) | 1.86 | 1 | 0.173 |
|  | Women | 129 | (37.7) | 16 | (50.0) |  |  |  |
| Ethnicity | |  |  |  |  |  |  |  |
|  | White British | 84 | (24.6) | 8 | (28.6) | 3.15 | 5 | 0.677 |
|  | White non-British | 42 | (12.3) | 4 | (14.3) |  |  |  |
|  | Black Caribbean | 58 | (17.0) | 2 | (7.1) |  |  |  |
|  | Black African | 88 | (25.7) | 6 | (21.4) |  |  |  |
|  | Asian (all) | 20 | (5.9) | 3 | (10.7) |  |  |  |
|  | Other | 50 | (14.6) | 5 | (17.9) |  |  |  |
| Diagnosis | |  |  |  |  |  |  |  |
|  | Schizophrenia | 172 | (51.2) | 8 | (53.3) | 3.21 | 2 | 0.201 |
|  | Other non-affective psychosis† | 83 | (24.1) | 6 | (40.0) |  |  |  |
|  | Affective psychosis | 81 | (24.7) | 1 | (6.7) |  |  |  |

Note. CECA, Childhood Experience of Care and Abuse interview.

† Includes schizoaffective disorder, delusional disorder, and psychosis NOS (which includes 23 with insufficient information to derive an OPCRIT diagnosis)

**Supplementary Table 4.** Main effects for each type of childhood adversity, by diagnosis.

|  | | Controls  n = 297†  n (%*) | | Cases, Non-Affective  n = 242†  n (%*) | | Cases, Affective  n = 78†  n (%*) | |
| --- | --- | --- | --- | --- | --- | --- | --- |
| Household Discord** | |  |  |  |  |  |  |
|  | No | 184 | (62.2) | 107 | (54.0) | 30 | (48.4) |
|  | Yes | 112 | (37.8) | 91 | (46.0) | 32 | (51.6) |
|  | Adj. Odds Ratio‡ | 1.00 | | 1.56 | | 1.88 | |
|  | 95% CI | - | | 1.03-2.37 | | 1.06-3.35 | |
| Psychological Abuse*** | |  |  |  |  |  |  |
|  | No | 203 | (95.9) | 169 | (85.8) | 53 | (85.5) |
|  | Yes | 12 | (4.1) | 28 | (14.2) | 9 | (14.5) |
|  | Adj. Odds Ratio‡ | 1.00 | | 4.00 | | 4.07 | |
|  | 95% CI | - | | 1.79-8.96 | | 1.46-11.30 | |
| Physical Abuse^ | |  |  |  |  |  |  |
|  | No | 234 | (79.1) | 147 | (63.9) | 46 | (66.7) |
|  | Yes | 62 | (20.9) | 83 | (36.1) | 23 | (33.3) |
|  | Adj. Odds Ratio‡ | 1.00 | | 2.27 | | 2.17 | |
|  | 95% CI | - | | 1.40-3.68 | | 1.10-4.26 | |
| Sexual Abuse^^ | |  |  |  |  |  |  |
|  | No | 273 | (93.5) | 188 | (86.7) | 54 | (79.4) |
|  | Yes | 19 | (6.5) | 29 | (13.3) | 14 | (20.6) |
|  | Adj. Odds Ratio‡ | 1.00 | | 2.24 | | 3.53 | |
|  | 95% CI | - | | 1.15-4.34 | | 1.61-7.73 | |
| Bullying^^^ | |  |  |  |  |  |  |
|  | No | 207 | (70.4) | 131 | (61.8) | 42 | (63.6) |
|  | Yes | 87 | (29.6) | 81 | (38.2) | 24 | (36.4) |
|  | Adj. Odds Ratio‡ | 1.00 | | 1.42 | | 1.38 | |
|  | 95% CI | - | | 0.94-2.16 | | 0.78-2.45 | |

Note (1): All analyses are weighted to account for oversampling of black Caribbean and black African controls

Note (2): 5 cases, no diagnosis

‡ Adjusted for age, sex, and ethnicity; * Percentages are for cases and controls with complete data; cases with childhood onset, excluded; ** 61 missing (1 control, 60 cases); *** 63 missing (2 controls, 61 cases); ^ 22 missing (1 control, 21 cases); ^^ 39 missing (5 controls, 34 cases); ^^^ 45 missing (3 controls, 42 cases)

**Supplementary Table 5.** Main effects for each type of childhood adversity, by sex.

|  | | Men | | | | | Women | | | | |
| --- | --- | --- | --- | --- | --- | --- | --- | --- | --- | --- | --- |
|  | | Controls  n = 153†  n (%*) | Cases  n = 201†  n (%*) | adj. OR‡ | 95% CI | p | Controls  n = 144†  n (%*) | Cases  n = 124†  n (%*) | adj. OR‡ | 95% CI | p |
| Household Discord** | |  |  |  |  |  |  |  |  |  |  |
|  | No | 100 (65.4) | 93 (55.7) | 1.00 | - | - | 84 (58.7) | 44 (45.4) | 1.00 | - | - |
|  | Yes | 53 (34.6) | 74 (44.3) | 1.47 | 0.86-2.50 | 0.156 | 59 (41.3) | 53 (54.6) | 2.20 | 1.23-3.97 | 0.008 |
| Psychological Abuse*** | |  |  |  |  |  |  |  |  |  |  |
|  | No | 147 (96.1) | 148 (88.1) | 1.00 | - | - | 136 (95.8) | 78 (81.3) | 1.00 | - | - |
|  | Yes | 6 (3.9) | 20 (11.9) | 4.63 | 1.64-13.02 | 0.004 | 6 (4.2) | 18 (18.7) | 4.36 | 1.53-12.45 | 0.006 |
| Physical Abuse‡ | |  |  |  |  |  |  |  |  |  |  |
|  | No | 116 (75.8) | 125 (65.8) | 1.00 | - | - | 118 (82.5) | 71 (62.3) | 1.00 | - | - |
|  | Yes | 37 (24.2) | 65 (34.2) | 1.66 | 0.91-3.00 | 0.096 | 25 (17.5) | 43 (37.7) | 3.54 | 1.63-7.69 | 0.001 |
| Sexual Abuse‡‡ | |  |  |  |  |  |  |  |  |  |  |
|  | No | 147 (97.4) | 168 (90.8) | 1.00 | - | - | 126 (89.4) | 77 (74.0) | 1.00 | - | - |
|  | Yes | 4 (2.6) | 17 (9.2) | 3.16 | 1.05-9.44 | 0.040 | 15 (10.6) | 27 (26.0) | 2.47 | 1.15-5.32 | 0.021 |
| Bullying^ | |  |  |  |  |  |  |  |  |  |  |
|  | No | 101 (66.5) | 110 (61.1) | 1.00 | - | - | 106 (74.7) | 66 (64.1) | 1.00 | - | - |
|  | Yes | 51 (33.5) | 70 (38.9) | 1.47 | 0.86-2.49 | 0.156 | 36 (25.3) | 37 (35.9) | 1.53 | 0.84-2.78 | 0.160 |

Note: All analyses are weighted to account for oversampling of black Caribbean and black African controls. CI, confidence interval; OR, odds ratio.

‡ Adjusted for age, sex, and ethnicity; * Percentages are for cases and controls with complete data; cases with childhood onset, excluded; ** 62 missing (1 control, 61 cases); *** 63 missing (2 controls, 61 cases); ‡ 22 missing (1 control, 21 cases); ‡‡ 40 missing (5 controls, 35 cases); ^ 45 missing (3 controls, 42 cases)

**Supplementary Table 6.** Main effects for each type of childhood adversity, by age group (at contact).

|  | | 18-29 years | | | | | 30-64 years | | | | |
| --- | --- | --- | --- | --- | --- | --- | --- | --- | --- | --- | --- |
|  | | Controls  n = 111†  n (%*) | Cases  n = 202†  n (%*) | adj. OR‡ | 95% CI | p | Controls  n = 186†  n (%*) | Cases  n = 123†  n (%*) | adj. OR‡ | 95% CI | p |
| Household Discord** | |  |  |  |  |  |  |  |  |  |  |
|  | No | 64 (57.7) | 84 (50.9) | 1.00 | - | - | 120 (64.9) | 53 (53.5) | 1.00 | - | - |
|  | Yes | 47 (42.3) | 81 (49.1) | 1.19 | 0.70-2.03 | 0.512 | 65 (35.1) | 46 (46.5) | 1.97 | 1.13-3.42 | 0.016 |
| Psychological Abuse*** | |  |  |  |  |  |  |  |  |  |  |
|  | No | 105 (94.6) | 140 (85.9) | 1.00 | - | - | 178 (96.7) | 86 (85.2) | 1.00 | - | - |
|  | Yes | 6 (5.4) | 23 (14.1) | 2.07 | 0.79-5.42 | 0.139 | 6 (3.3) | 15 (14.8) | 6.35 | 2.08-19.42 | 0.001 |
| Physical Abuse‡ | |  |  |  |  |  |  |  |  |  |  |
|  | No | 95 (86.4) | 127 (66.8) | 1.00 | - | - | 139 (74.7) | 69 (60.5) | 1.00 | - | - |
|  | Yes | 15 (13.6) | 63 (33.2) | 3.06 | 1.47-6.36 | 0.003 | 47 (25.3) | 45 (39.5) | 1.97 | 1.08-3.59 | 0.028 |
| Sexual Abuse‡‡ | |  |  |  |  |  |  |  |  |  |  |
|  | No | 100 (93.5) | 156 (86.2) | 1.00 | - | - | 173 (93.5) | 89 (82.4) | 1.00 | - | - |
|  | Yes | 7 (6.5) | 25 (13.8) | 1.84 | 0.71-4.74 | 0.207 | 12 (6.5) | 19 (17.6) | 2.31 | 1.06-5.06 | 0.036 |
| Bullying^ | |  |  |  |  |  |  |  |  |  |  |
|  | No | 75 (67.6) | 105 (58.7) | 1.00 | - | - | 132 (72.1) | 71 (68.3) | 1.00 | - | - |
|  | Yes | 36 (32.4) | 74 (41.3) | 1.63 | 0.95-2.83 | 0.076 | 51 (27.9) | 33 (31.7) | 1.26 | 0.72-2.21 | 0.420 |

Note: All analyses are weighted to account for oversampling of black Caribbean and black African controls. CI, confidence interval; OR, odds ratio.

‡ Adjusted for age, sex, and ethnicity; * Percentages are for cases and controls with complete data; cases with childhood onset, excluded; ** 64 missing (1 control, 63 cases); *** 64 missing (2 controls, 62 cases); ‡ 23 missing (1 control, 22 cases); ‡‡ 46 missing (5 controls, 41 cases); ^ 46 missing (3 controls, 43 cases)

**Supplementary Table 7.** Main effects for each type of childhood adversity (on restricted sample with data on all parent history of psychosis and social class).

|  | | Controls  n = 264  n (%*) | | Cases  n = 240  n (%*) | | adj. OR (1) † | 95% CI | p | adj. OR (2)‡ | 95% CI | p |
| --- | --- | --- | --- | --- | --- | --- | --- | --- | --- | --- | --- |
| Household Discord** | |  |  |  |  |  |  |  |  |  |  |
|  | No | 166 | (62.9) | 108 | (52.9) | 1.00 | - | - | 1.00 | - | - |
|  | Yes | 98 | (37.1) | 96 | (47.1) | 1.62 | 1.06-2.47 | 0.025 | 1.47 | 0.95-2.29 | 0.086 |
| Psychological Abuse*** | |  |  |  |  |  |  |  |  |  |  |
|  | No | 250 | (95.4) | 177 | (85.9) | 1.00 | - | - | 1.00 | - | - |
|  | Yes | 12 | (4.6) | 29 | (14.1) | 3.44 | 1.51-7.84 | 0.003 | 3.02 | 1.31-7.00 | 0.010 |
| Physical Abuse‡ | |  |  |  |  |  |  |  |  |  |  |
|  | No | 207 | (78.7) | 154 | (67.0) | 1.00 | - | - | 1.00 | - | - |
|  | Yes | 56 | (21.3) | 76 | (33.0) | 1.64 | 0.99-2.71 | 0.055 | 1.34 | 0.78-2.32 | 0.294 |
| Sexual Abuse‡‡ | |  |  |  |  |  |  |  |  |  |  |
|  | No | 243 | (93.4) | 189 | (86.2) | 1.00 | - | - | 1.00 | - | - |
|  | Yes | 17 | (6.6) | 31 | (13.8) | 2.49 | 1.23-5.03 | 0.011 | 2.43 | 1.15-5.12 | 0.020 |
| Bullying^ | |  |  |  |  |  |  |  |  |  |  |
|  | No | 184 | (69.7) | 129 | (58.3) | 1.00 | - | - | 1.00 | - | - |
|  | Yes | 79 | (30.3) | 88 | (41.8) | 1.50 | 0.99-2.27 | 0.054 | 1.42 | 0.92-2.21 | 0.114 |

Note: All analyses are weighted to account for oversampling of black Caribbean and black African controls. CI, confidence interval; OR, odds ratio.

† Adjusted for age, sex, and ethnicity; ‡ Adjusted for age, sex, and ethnicity, parental psychosis, parental social class; * Percentages are for cases and controls with complete data; cases with childhood onset, excluded; ** 36 missing (36 cases); *** 36 missing (2 controls, 34 cases); ‡ 11 missing (1 control, 10 cases); ‡‡ 24 missing (4 controls, 20 cases); ^ 24 missing (1 controls, 23 cases)

**Supplementary Table 8.** Main effects for each type of childhood adversity (on sample for whom there was no doubt in CECA ratings).

|  | | Controls  n = 280†  n (%*) | | Cases  n = 153†  n (%*) | | unadj. OR | 95% CI | p | adj. OR‡ | 95% CI | p |
| --- | --- | --- | --- | --- | --- | --- | --- | --- | --- | --- | --- |
| Household Discord** | |  |  |  |  |  |  |  |  |  |  |
|  | No | 178 | (63.8) | 71 | (47.7) | 1.00 | - | - | 1.00 | - | - |
|  | Yes | 101 | (36.2) | 78 | (52.4) | 2.02 | 1.32-3.08 | 0.001 | 2.10 | 1.34-3.28 | 0.001 |
| Psychological Abuse*** | |  |  |  |  |  |  |  |  |  |  |
|  | No | 267 | (95.7) | 127 | (84.1) | 1.00 | - | - | 1.00 | - | - |
|  | Yes | 12 | (4.3) | 24 | (15.9) | 4.54 | 2.09-9.88 | <0.001 | 4.21 | 1.98-8.94 | <0.001 |
| Physical Abuse*** | |  |  |  |  |  |  |  |  |  |  |
|  | No | 220 | (78.9) | 98 | (64.9) | 1.00 | - | - | 1.00 | - | - |
|  | Yes | 59 | (21.1) | 53 | (35.1) | 2.29 | 1.43-3.67 | 0.001 | 2.43 | 1.40-4.22 | 0.002 |
| Sexual Abuse^ | |  |  |  |  |  |  |  |  |  |  |
|  | No | 258 | (93.5) | 126 | (86.3) | 1.00 | - | - | 1.00 | - | - |
|  | Yes | 18 | (6.5) | 20 | (13.7) | 2.30 | 1.11-4.74 | 0.025 | 2.18 | 1.08-4.39 | 0.030 |
| Bullying^^ | |  |  |  |  |  |  |  |  |  |  |
|  | No | 192 | (69.1) | 92 | (60.9) | 1.00 | - | - | 1.00 | - | - |
|  | Yes | 86 | (30.9) | 59 | (39.1) | 1.61 | 1.05-2.49 | 0.031 | 1.25 | 0.80-1.96 | 0.324 |

Note: All analyses are weighted to account for oversampling of young and minority ethnic controls. CECA, Childhood Experience of Care and Abuse interview. CI, confidence interval; OR, odds ratio.

† Adjusted for age, sex, and ethnicity; ‡ Adjusted for age, sex, and ethnicity, parental psychosis, cannabis use, parental social class; * Percentages are for cases and controls with complete data; cases with childhood onset, excluded; ** 5 missing (1 control, 4 cases); *** 3 missing (2 controls, 1 cases); ^ 11 missing (4 controls, 7 cases); ^^ 4 missing (2 controls, 2 cases)

**Supplementary Table 9.** Main effects for each type of childhood adversity, by age of first occurrence. (See Table 2)

|  | | Controls  n = 297†  n (%*) | | Cases  n = 325†  n (%*) | | unadj. OR | 95% CI | p | adj. OR†‡ | 95% CI | p |
| --- | --- | --- | --- | --- | --- | --- | --- | --- | --- | --- | --- |
| Household Discord** | |  |  |  |  |  |  |  |  |  |  |
|  | No | 184 | (62.4) | 142 | (51.8) | 1.00 | - | - | 1.00 | - | - |
|  | 0-11 yrs | 83 | (28.1) | 105 | (38.3) | 1.63 | 1.10-2.41 | 0.014 | 1.68 | 1.10-2.55 | 0.016 |
|  | 12-16 yrs | 28 | (9.5) | 27 | (9.9) | 1.34 | 0.72-2.41 | 0.350 | 1.70 | 0.83-3.48 | 0.170 |
| Psychological Abuse*** | |  |  |  |  |  |  |  |  |  |  |
|  | No | 283 | (95.9) | 236 | (84.9) | 1.00 | - | - | 1.00 | - | - |
|  | 0-11 yrs | 9 | (3.1) | 34 | (12.2) | 4.16 | 1.80-9.54 | 0.001 | 4.15 | 1.65-10.42 | 0.002 |
|  | 12-16 yrs | 3 | (1.0) | 8 | (2.9) | 4.31 | 1.04-17.88 | 0.044 | 3.15 | 0.80-12.36 | 0.100 |
| Physical Abuse‡ | |  |  |  |  |  |  |  |  |  |  |
|  | No | 231 | (79.4) | 197 | (64.4) | 1.00 | - | - | 1.00 | - | - |
|  | 0-11 yrs | 52 | (17.9) | 86 | (28.1) | 2.09 | 1.35-3.22 | 0.001 | 2.07 | 1.24-3.44 | 0.005 |
|  | 12-16 yrs | 8 | (2.8) | 23 | (7.5) | 4.32 | 1.77-10.52 | 0.001 | 3.56 | 1.42-8.93 | 0.007 |
| Sexual Abuse‡‡ | |  |  |  |  |  |  |  |  |  |  |
|  | No | 273 | (93.2) | 245 | (82.5) | 1.00 | - | - | 1.00 | - | - |
|  | 0-11 yrs | 15 | (5.1) | 33 | (10.7) | 2.14 | 1.06-4.31 | 0.033 | 2.42 | 1.21-4.82 | 0.012 |
|  | 12-16 yrs | 5 | (1.7) | 21 | (6.8) | 5.77 | 2.09-15.90 | 0.001 | 6.39 | 1.68-24.29 | 0.006 |
| Bullying^ | |  |  |  |  |  |  |  |  |  |  |
|  | No | 207 | (70.2) | 179 | (60.7) | 1.00 | - | - | 1.00 | - | - |
|  | 0-11 yrs | 56 | (19.0) | 63 | (21.4) | 1.41 | 0.91-2.19 | 0.128 | 1.19 | 0.75-1.90 | 0.463 |
|  | 12-16 yrs | 32 | (10.8) | 53 | (17.8) | 2.00 | 1.19-3.36 | 0.008 | 1.75 | 1.05-2.96 | 0.033 |

Note: All analyses are weighted to account for oversampling of black Caribbean and black African controls. CI, confidence interval; OR, odds ratio.

‡ Adjusted for age, sex, and ethnicity; * Percentages are for cases and controls with complete data; ** 52 missing (1 control, 51 cases); *** 49 missing (2 controls, 47 cases); ‡ 20 missing (1 control, 19 cases); ‡‡ 31 missing (5 controls, 26 cases); ^ 33 missing (3 controls, 30 cases)

**Supplementary Table 10.** Main effects for each type of childhood adversity, by frequency.

|  | | Controls  n = 297†  n (%*) | | Cases  n = 325†  n (%*) | | unadj. OR | 95% CI | p | adj. OR†‡ | 95% CI | p |
| --- | --- | --- | --- | --- | --- | --- | --- | --- | --- | --- | --- |
| Household Discord** | |  |  |  |  |  |  |  |  |  |  |
|  | No | 184 | (62.2) | 137 | (53.1) | 1.00 | - | - | 1.00 | - | - |
|  | Less than monthly | 12 | (4.1) | 28 | (10.9) | 3.75 | 1.75-8.02 | 0.001 | 3.36 | 1.39-8.09 | 0.007 |
|  | Monthly | 100 | (33.8) | 93 | (36.1) | 1.27 | 0.87-1.86 | 0.216 | 1.40 | 0.92-2.11 | 0.114 |
| Psychological Abuse*** | |  |  |  |  |  |  |  |  |  |  |
|  | No | 283 | (95.9) | 226 | (86.3) | 1.00 | - | - | 1.00 | - | - |
|  | Less than monthly | 3 | (1.0) | 5 | (1.9) | 1.61 | 0.35-7.46 | 0.544 | 1.18 | 0.31-4.43 | 0.805 |
|  | Monthly | 9 | (3.1) | 31 | (11.8) | 5.38 | 2.41-11.98 | <0.001 | 5.58 | 2.25-13.82 | <0.001 |
| Physical Abuse‡ | |  |  |  |  |  |  |  |  |  |  |
|  | No | 233 | (79.0) | 192 | (66.7) | 1.00 | - | - | 1.00 | - | - |
|  | Less than monthly | 26 | (8.8) | 40 | (13.9) | 1.97 | 1.13-3.43 | 0.017 | 2.40 | 1.27-4.52 | 0.007 |
|  | Monthly | 36 | (12.2) | 56 | (19.4) | 2.26 | 1.36-3.77 | 0.002 | 1.87 | 1.04-3.35 | 0.036 |
| Sexual Abuse‡‡ | |  |  |  |  |  |  |  |  |  |  |
|  | No | 273 | (93.8) | 245 | (86.0) | 1.00 | - | - | 1.00 | - | - |
|  | Less than monthly | 11 | (3.8) | 31 | (10.9) | 3.53 | 1.66-7.52 | 0.001 | 3.38 | 1.52-7.53 | 0.003 |
|  | Monthly | 7 | (2.4) | 9 | (3.2) | 1.58 | 0.56-4.47 | 0.390 | 1.42 | 0.55-3.71 | 0.469 |
| Bullying^ | |  |  |  |  |  |  |  |  |  |  |
|  | No | 203 | (70.0) | 175 | (63.0) | 1.00 | - | - | 1.00 | - | - |
|  | Less than monthly | 9 | (3.1) | 28 | (10.1) | 4.72 | 2.09-10.65 | <0.001 | 4.01 | 1.73-9.37 | 0.001 |
|  | Monthly | 78 | (26.9) | 75 | (26.9) | 1.24 | 0.83-1.84 | 0.297 | 1.12 | 0.74-1.70 | 0.588 |

Note: All analyses are weighted to account for oversampling of black Caribbean and black African controls. CI, confidence interval; OR, odds ratio.

‡ Adjusted for age, sex, and ethnicity; * Percentages are for cases and controls with complete data; ** 68 missing (1 control, 67 cases); *** 69 missing (6 controls, 63 cases); ‡ 43 missing (6 control, 37 cases); ‡‡ 50 missing (10 controls, 40 cases); ^ 53 missing (11 controls, 42 cases)

**Supplementary Table 11.** Associations between each childhood adversity.

(a) Cases

|  | H’hold Discord | Psych. Abuse | Phys. Abuse | Sexual Abuse | Bullying |
| --- | --- | --- | --- | --- | --- |
| H’hold Discord | - |  |  |  |  |
| Psych. Abuse | x^2^ 33.7  p < 0.001 | - |  |  |  |
| Phys. Abuse | x^2^ 9.8  p 0.002 | x^2^ 30.8  p < 0.001 | - |  |  |
| Sexual Abuse | x^2^ 6.1  p 0.14 | x^2^ 13.7  p < 0.001 | x^2^ 14.6  p < 0.001 | - |  |
| Bullying | x^2^ 4.2  p 0.039 | x^2^ 7.6  p 0.006 | x^2^ 3.6  p 0.059 | x^2^ 5.6  p 0.018 | - |

(b) Controls

|  | H’hold Discord | Psych. Abuse | Phys. Abuse | Sexual Abuse | Bullying |
| --- | --- | --- | --- | --- | --- |
| H’hold Discord | - |  |  |  |  |
| Psych. Abuse | x^2^ 11.1  p 0.001 | - |  |  |  |
| Phys. Abuse | x^2^ 9.9  p 0.002 | x^2^ 22.4  p < 0.001 | - |  |  |
| Sexual Abuse | x^2^ 0.9  p 0.339 | x^2^ 0.7  p 0.163 | x^2^ 12.4  p < 0.001 | - |  |
| Bullying | x^2^ 7.5  p 0.006 | x^2^ 0.8  p 0.358 | x^2^ 3.4  p 0.064 | x^2^ 0.8  p 0.362 | - |

**Supplementary Table 12.** Number of adversities and psychotic disorder.

|  | | Controls  n = 297†*  n (%) | | Cases  n = 325†*  n (%) | | Adj. OR ‡ | 95% CI | p |
| --- | --- | --- | --- | --- | --- | --- | --- | --- |
| Number of adversities | |  |  |  |  |  |  |  |
|  | 0 | 115 | (39.0) | 58 | (20.3) | 1.00 | - | - |
|  | 1 | 99 | (33.6) | 114 | (39.9) | 2.11 | 1.33-3.35 | 0.002 |
|  | 2 | 58 | (19.7) | 62 | (21.7) | 2.63 | 1.53-4.52 | <0.001 |
|  | 3 | 16 | (5.4) | 30 | (10.5) | 3.46 | 1.47-8.14 | 0.004 |
|  | 4 or 5 | 7 | (2.4) | 22 | (7.7) | 5.65 | 2.23-14.28 | 0.001 |

Note: All analyses are weighted to account for oversampling of black Caribbean and black African controls. CI, confidence interval. OR, odds ratio.

* 41 missing (2 controls; 39 cases); ‡ Adjusted for age, gender, and ethnicity.

**Supplementary Table 13.** Main effects for each type of childhood adversity, by severity. (See Table 4)

|  | | Controls  n = 297†  n (%*) | | Cases  n = 325†  n (%*) | | unadj. OR | 95% CI | p | adj. OR†‡ | 95% CI | p |
| --- | --- | --- | --- | --- | --- | --- | --- | --- | --- | --- | --- |
| Household Discord** | |  |  |  |  |  |  |  |  |  |  |
|  | No | 184 | (62.2) | 137 | (51.9) | 1.00 | - | - | 1.00 | - | - |
|  | Moderate | 57 | (19.3) | 33 | (12.5) | 0.80 | 0.48-1.33 | 0.396 | 0.87 | 0.50-1.52 | 0.623 |
|  | Marked | 32 | (10.8) | 27 | (10.2) | 1.19 | 0.66-2.13 | 0.558 | 1.22 | 0.64-2.31 | 0.552 |
|  | Violence | 23 | (7.8) | 67 | (25.4) | 3.99 | 2.23-7.11 | <0.001 | 4.40 | 2.37-8.16 | <0.001 |
| Psychological Abuse*** | |  |  |  |  |  |  |  |  |  |  |
|  | No | 283 | (95.9) | 226 | (85.6) | 1.00 | - | - | 1.00 | - | - |
|  | Moderate | 9 | (3.1) | 23 | (8.7) | 3.26 | 1.39-7.66 | 0.007 | 3.45 | 1.35-8.82 | 0.010 |
|  | Marked | 3 | (1.0) | 15 | (5.7) | 8.24 | 2.22-30.62 | 0.002 | 5.60 | 1.47-21.36 | 0.012 |
| Physical Abuse‡ | |  |  |  |  |  |  |  |  |  |  |
|  | No | 234 | (79.1) | 195 | (64.4) | 1.00 | - | - | 1.00 | - | - |
|  | Moderate | 53 | (17.9) | 86 | (28.4) | 2.12 | 1.39-3.25 | 0.001 | 2.07 | 1.27-3.39 | 0.004 |
|  | Marked | 9 | (3.0) | 22 | (7.3) | 4.36 | 1.92-9.94 | <0.001 | 3.97 | 1.59-9.89 | 0.003 |
| Sexual Abuse‡‡ | |  |  |  |  |  |  |  |  |  |  |
|  | No | 273 | (93.5) | 245 | (84.8) | 1.00 | - | - | 1.00 | - | - |
|  | Moderate | 10 | (3.4) | 11 | (3.8) | 1.24 | 0.50-3.10 | 0.640 | 1.58 | 0.65-3.88 | 0.313 |
|  | Marked | 9 | 3.1) | 33 | (11.4) | 4.06 | 1.69-9.76 | 0.002 | 3.73 | 1.63-8.54 | 0.002 |
| Bullying^ | |  |  |  |  |  |  |  |  |  |  |
|  | No | 207 | (70.4) | 176 | (62.2) | 1.00 | - | - | 1.00 | - | - |
|  | Moderate | 68 | (20.1) | 68 | (24.0) | 1.35 | 0.89-2.04 | 0.156 | 1.27 | 0.82-1.95 | 0.284 |
|  | Marked | 19 | (6.5) | 39 | (13.8) | 2.52 | 1.36-4.67 | 0.003 | 1.91 | 1.00-3.64 | 0.048 |

Note: All analyses are weighted to account for oversampling of black Caribbean and black African controls. CI, confidence interval; OR, odds ratio.

‡ Adjusted for age, sex, and ethnicity; * Percentages are for cases and controls with complete data; cases with childhood onset, excluded; ** 62 missing (1 control, 61 cases); *** 63 missing (2 controls, 61 cases); ‡ 23 missing (1 control, 22 cases); ‡‡ 41 missing (5 controls, 36 cases); ^ 45 missing (3 controls, 42 cases)
