# Supplementary material for: Threat, hostility and violence in childhood and later psychotic disorder: population-based case–control study
Source: Br J Psychiatry. 2020 Oct;217(4):575–82. doi: 10.1192/bjp.2020.133 (PMC7525109; doi:10.1192/bjp.2020.133)
Supplement: Supplementary file 1 [file S0007125020001336sup001.zip › S0007125020001336sup002.docx]

**Supplementary Materials (2) Appendix**

**Control Selection and Recruitment**

A population-based and demographically representative sample of controls resident in our catchment areas, aged 18-64 years, and without a current or past history of psychotic disorder was recruited using a mixture of quota and random sampling. First, quotas were set for gender, age group, and ethnic group. The quotas for each group were set to ensure recruitment of a sample of controls that reflected the demographic profile, based on the 2011 UK Census, of the local population and that included a sufficient number of controls from black Caribbean and black African groups for potential analyses by ethnic group. Second, two sampling frames were used to fill these quotas: a) the UK postal address file (PAF) and b) general practitioner (GP) lists. The Royal Mail Small Users PAF (16) provides a list of all households in the UK. We used this to randomly select addresses within our catchment areas. The selected addresses were sent letters of invitation to take part and, then, at least two weeks later, each address was visited on at least four separate occasions at different times of the day (morning, afternoon, and evening) and on different days of the week (including weekends). Residents were given written and verbal information concerning the study and were asked whether anyone in the household might be eligible and interested in taking part. If all potential controls within the household refused, or no members were eligible, then the next address on the PAF list was visited. A total of 695 letters were sent; 326 potential controls were identified; and 133 (44.2%) were selected (i.e., fit one of quotas), recruited, and assessed. In addition, in the catchment areas, 12 GP surgeries were randomly selected and, from the lists of each of these, 3600 individuals who met the inclusion criteria for controls were randomly selected and sent letters of invitation to take part. A total of 515 responded to the invitation and 168 (32.6%) were selected (i.e., fit one of quotas), recruited, and assessed.
